# Maximizing the benefits of using biosimilars in Egypt

**DOI:** 10.1186/s40545-023-00581-w

**Published:** 2023-06-26

**Authors:** Ahmad Nader Fasseeh, Baher Elezbawy, Kareem Ahmed El-Fass, Mary GamaI, Ahmed Seyam, Noha Hayek, Nagwan Abdel Rahman, Sohir Abdelhamid, Nader Fasseeh, Amr Shafik Saad, Ahmed Elagamy, Akram Mahmoud, Amal Samir Sedrak, Khaled Elshazly, Mariam Eldebeiky, Monica Talaat, Noura Maher Mohamed, Rania Ahmed Abdelaziz, Remonda Refaat, Shaimaa Akeel, Sherif Abaza, Zoltán Kaló

**Affiliations:** 1 Syreon Middle East, Alexandria, Egypt; 2 Faculty of Pharmacy, Alexandria University, Alexandria, Egypt; 3 Egyptian Authority for Unified Procurement, Medical Supply and Technology Management, Cairo, Egypt; 4 Universal Health Insurance Authority, Cairo, Egypt; 5 Theodor Bilharz Research Institute, Giza, Egypt; 6 Health Insurance Organization, Cairo, Egypt; 7 Egyptian Parliament Health Sector, Cairo, Egypt; 8 Faculty of Medicine, Alexandria University, Alexandria, Egypt; 9 Faculty of Medicine, Ain Shams University, Cairo, Egypt; 10 Faculty of Medicine, Cairo University, Cairo, Egypt; 11 Presidential Program for Women’s Health, Cairo, Egypt; 12 Egyptian Drug Authority, Cairo, Egypt; 13 Semmelweis University, Budapest, Hungary; 14 Syreon Research Institute, Budapest, Hungary

**Keywords:** Biosimilars, Health policy, Healthcare sustainability, Patient access, Reimbursement

## Abstract

**Background:**

Biosimilars constitute a pathway for sustainable financing of healthcare systems in the era of expensive biologics. However, such a pathway is not free of challenges. Since the biosimilars market is expanding in Egypt, there is an urgent need for a policy framework to optimize their use and diffusion in the market. We aim to characterize a national framework based on the experiences of other countries and consultation with local experts.

**Methods:**

A narrative literature review was conducted to identify biosimilars’ policy elements worldwide. A workshop was organized with experts to discuss the narrative review findings and create consensus on recommendations.

**Results:**

The narrative literature review highlighted the need for biosimilar policy actions in four areas: market authorization, pricing, reimbursement, and uptake. Eighteen experts representing the Egyptian healthcare authorities attended the workshop. The most significant conclusions from the workshop included setting the price of the biosimilar at 30–40% less than its originator’s price and establishing financing protocols, in which the more expensive biologics with significant price premiums should be excluded from the formulary.

**Conclusions:**

A summarized national framework policy recommendation for biosimilars was created by local experts from the main public healthcare entities in Egypt. These recommendations coincide with the international policies adopted across different countries that aim to improve patient access while sustaining health expenditure.

**Supplementary Information:**

The online version contains supplementary material available at 10.1186/s40545-023-00581-w.

## Background

Biological therapies offer therapeutic advantages and improve patients’ outcomes in many disease areas compared to previous simple molecules [1–3]. However, they represent a huge burden on healthcare systems and patients due to their high cost [4]. Consequently, patients’ access to these medications can be limited. Healthcare systems usually face pressure to reimburse these new biologics to maximize the patient’s health gain despite the limited resources available [5].

Biosimilars are products similar to the innovator biologics in terms of biological activity, safety, and efficacy [6], yet they are not identical [7]. The uptake of biosimilars within the healthcare systems can help reduce pharmaceutical expenditure in specific categories without compromising health benefits. Adopting biosimilars could help maintain the sustainability of the healthcare system in part—due to allocative efficiency—and help improve health outcomes in the long term [8, 9].

In Egypt, the health system has been challenged by underinvestment and inefficiencies [10]. In 2019, the current health expenditure (CHE) was 4.74% of the Gross Domestic Product (GDP), which is less than the average of low and middle-income countries, 5.32% [11]. The pharmaceutical expenditure represented approximately 34% of the CHE [12].

The Universal Health Insurance Law introduced in 2018 aimed to overcome these inefficiencies and promote health equity through enhanced patients’ access to high-value medical care. The law includes articles that aim to ensure the availability of a better healthcare system through providing medical insurance to all citizens through a quality-controlled healthcare system according to present quality standards [12].

Egypt is a country with a population of approximately 108 million [13]. The healthcare financing system in Egypt is complex, involving multiple public and private providers. The mean total health expenditure in Egypt is 222 billion EGP [12], and the mean pharmaceutical expenditure ranges from 40 to 67 billion. The Ministry of Health and Population (MoHP) is a key player, where all uninsured citizens are eligible to use MoHP curative services and can also benefit from the Program for Treatment at the Expense of the State (PTES). People are insured by public organizations such as Health Insurance Organization (HIO) or private insurance companies. HIO covers approximately 55% of the population [12]. There are other public organizations that provide health services to particular segments of the population, such as the Ministry of higher education, and Ministry of Defense, and the Ministry of Interior [12]. For some interventions, there are co-payments (which are usually capped) according to the bylaws of each organization. Also, according to the bylaws of each organization, some medications require pre-authorization.

In Egypt, there are two drug formularies used to reimburse medications. First, The Health Insurance Organization (HIO) formulary lists medications by their International Nonproprietary Names (INNs) and does not have any reimbursement criteria. This formulary applies only to outpatients. Second, The Universal Health Insurance Authority (UHIA) formulary lists medications by their INNs and brand names. Innovative medicines are listed in the UHIA formulary after undergoing a Health Technology Assessment (HTA) process. In the UHIA formulary, in the case of multisource products, the generics with the least price (preferred generic) are fully reimbursed, while higher-price generics and brands are partially reimbursed, and patients pay a co-payment (the difference between the price of the preferred generics and the dispensed medicine). This formulary applies to inpatients and outpatients. It is noteworthy that the Program for Treatment at the Expense of the State (PTES) does not have a formulary.

Although the out-of-pocket payments represent the main health financing source in Egypt (63%), the governmental health expenditure is substantial (111 billion EGP) [12]. Also, when it comes to expensive medications (e.g., biologics), approximately 80% of the expenditure on expensive medications is publicly funded.

The biosimilar market in Egypt is expanding. There are more than 55 biosimilar products currently on the market [14]. Additionally, The Egyptian International Pharmaceutical Industries Company (EIPICO)—a giant Egyptian pharmaceutical industry firm- is looking to start operating its Biosimilar Project in 2023 [15]. In 2020, The Egyptian Drug Authority (EDA) has released an updated guideline for biosimilar registration [16]. It covers the registration and market authorization process details, comparability exercises, safety, and efficacy documents. Nevertheless, it does not include details of the policy framework for pricing, reimbursement, or uptake. These policies are required for biosimilars’ sustainability [17].

In the current study, we aim to propose a policy framework for pricing, reimbursement, and biosimilar uptake in Egypt. The results of this study will provide specific recommendations to be made by decision-makers in Egypt for the effective utilization of biosimilars. It can also help other countries that aim to create or adjust their biosimilars’ policy frameworks.

## Methods

To optimize the regulatory framework in Egypt, we adopted a three-step approach. First, we conducted a narrative literature review [18] to understand the current biosimilars regulatory framework (pricing, reimbursement, and uptake) in Egypt and in other countries. After that, a workshop was conducted, which started by disseminating a survey that intended to answer questions about policies related to pricing, reimbursement, and uptake of biosimilars in Egypt. After that, during the workshop, which involved experts from several public healthcare entities in Egypt, the survey findings were discussed, and participants shared their recommendations for optimizing the biosimilar policy framework.

### Narrative literature review

A narrative non-systematic literature review was conducted to explore biosimilars' specific policy frameworks in different countries and in Egypt. The following keywords were used for searching the literature: (biosimilar, registration, market authorization, policies, reimbursement, HTA, pricing, switching, guidelines). We searched PubMed, Google Scholar, and Google search engines for relevant studies or documents. In addition, the FDA (Food and Drug Administration) and EMA (European Medicines Agency) websites were searched for relevant information on biosimilar policies. Also, snowball searching was used. The search was conducted in November 2021. Studies and reports were included only if they included data about any of the keywords mentioned above regarding biosimilar policy. English studies only were selected, and there was no date restriction for the studies included.

### Survey preparation

We designed a survey to collect the stakeholders’ opinions about biosimilar utilization elements (pricing, reimbursement, uptake, and diffusion in the healthcare system) in Egypt and ask them about their recommendations for optimizing biosimilars use. The narrative literature review results guided us in formulating the survey questions. The survey questions aimed to formulate the utilization elements that are not currently available in Egypt but are commonly available in other countries or jurisdictions.

The survey was designed by AF, BE, MG, and KE and then reviewed by ZK to ensure relevance and non-ambiguity. It included 15 questions grouped into four domains: overall perception about biosimilars, pricing, reimbursement, and uptake. The stakeholders responded to the survey during a live workshop.

### Workshop

The workshop was held in Alexandria, Egypt, on the 7th of December 2021 to evaluate biosimilar policies in Egypt, assess the gaps compared to experiences from other countries, and propose a national policy framework for biosimilars.

The stakeholders attending the workshop were selected using a convenience sampling method. They were selected based on pre-determined inclusion criteria, being influential stakeholders in the Egyptian healthcare system, and having a good understanding of health technology assessment, pricing, reimbursement, and pharmaceutical policies. We ensured having at least one member from each major public entity related to healthcare in Egypt.

At the beginning of the workshop, the stakeholders were presented with the narrative literature review results. Then, they filled in the survey questions through an online anonymous voting system (Mentimeter^©^) using their smartphones. The voting system automatically aggregated the votes and created a bar chart for each question illustrating the frequency of votes for each choice (see Additional file 1). These charts were then presented to the stakeholders attending the workshop to discuss the results of the aggregated responses. Finally, all stakeholders had an open discussion that aimed to shape the future and reach a consensus on recommendations for the biosimilar policy framework in Egypt.

### Ethical approval

At the beginning of the survey, respondents were asked if they agreed to participate in the study. All of them agreed.

### Statistical analysis and formulating recommendations

Simple statistical analyses were conducted for the survey responses to show the percentage of responses for each choice option based on the number of responders to each question. During the discussion section of the workshop, we took notes of the recommendations that were agreed on. These were formulated into final recommendations after the workshop and were further validated by the survey responders via emails. The survey respondents then suggested some minor phrasing edits to the recommendations. These edits were adjusted, then all respondents approved a final version of the recommendations.

## Results

### Narrative literature review findings

When we explored the literature, we grouped the related findings together into domains. The identified data were related to four domains: market authorization, pricing, reimbursement, and uptake. There were no results pertinent to Egypt’s biosimilar policy framework except for the market authorization.

#### Market authorization

The market authorization domain in Egypt was well covered in the Egyptian Drug Authority (EDA) guidelines published in 2012 and updated in 2020 [16]. The EDA guidelines focused on comparability exercises required to ensure the safety and efficacy of biosimilars. In addition, it addressed the necessary conditions for the extrapolation of biosimilar indications. Nevertheless, there is insufficient data about the recommended timing of registration application admission of biosimilars, whether before or after the originator patency expiry.

#### Pricing

Biosimilars are usually priced according to internal or external reference pricing systems or price negotiations [19–21]. In certain jurisdictions, introducing a new biosimilar may lead to a mandatory reduction in the originator's price. Pricing of subsequent biosimilars may be subjected to more discounts from the originator prices [19, 20].

#### Reimbursement

Adopting biosimilars may lead to budget-saving, allowing the payers to expand their reimbursement capacities to more innovative products using the saved budget (headroom for innovation) [22, 23]. When a biosimilar is approved, it can be integrated into existing treatment protocols as a first-line or a second-line option [24, 25]. It is recommended in some countries that biosimilars are used as the first line, and the originator biologics are used when biosimilars fail [26]. In some jurisdictions, mandatory switching to biosimilars for patients on originators was introduced [27, 28]. Furthermore, some countries remove the biosimilar product from the positive list if its price is 30% higher than that of the reference product [29]. Some countries apply policies to maintain the biosimilar in the formulary for a predefined period under certain conditions [30].

Countries differ in the HTA techniques they adopt for biosimilars. Some adopt full HTA (HTA reports that include a description of the health technology, evaluation of its safety, effectiveness, cost-effectiveness, financial impact, organizational considerations, and a comprehensive systematic literature review conducted) [31]; other countries recommend only cost-effectiveness studies or budget impact analyses, while some countries require only cost-minimization analysis for biosimilars reimbursement [32]. Some countries apply health economic evaluation if the reimbursed indication is significantly expanded [33].

Some safety issues may arise when biosimilars are marketed widely; accordingly, different pharmacovigilance frameworks are implemented worldwide. For example, some countries provide incentives to establish registries for biosimilar products to record safety and effectiveness issues based on real-world data [5], while awareness campaigns for the public and physicians are recommended in other regions to encourage the use of biosimilars [34].

#### Biosimilars’ uptake

Prescribers and patients are core stakeholders in biosimilar use [35]. A lack of policies addressing their concerns and enhancing their acceptance of biosimilars can lead to the failure of biosimilars adoption policies [26]. Policies to enhance prescribers' acceptance of biosimilars include dissemination of educational materials, and biosimilars prescribing guidelines, and highlighting the benefits of biosimilars adoption to the healthcare system [35]. Also, educating the prescribers about the real-world evidence about switching to biosimilars to mitigate the excessive fear of immunogenicity [35]. Some countries apply financial incentives and penalties to encourage the use of biosimilars as first-line therapy [24].

Enhancing patients’ acceptance of biosimilars is necessary; they are the real users of these medicines. Policies to help patients accept biosimilars include educating the patients about the benefits of biosimilars, including better access to innovative therapies [34]. Some countries apply co-payments for patients to increase the uptake of biosimilars [24].

### Survey design

We designed the survey based on the practices and policies related to biosimilars observed globally. Since the market authorization domain is well covered in EDA guidelines, our survey did not include questions about it. The survey included questions in the other three domains (pricing, reimbursement, and uptake) in addition to an introductory domain about the overall perception of the experts about biosimilars. Additionally, we added a domain about the overall perception of biosimilars to include general introductory questions for the local stakeholders’ perception of biosimilars. We created the survey questions based on the studies and data we identified in the narrative literature review. We used closed-ended questions to make the survey easier to answer and easily analyzable.

### Workshop results

Eighteen stakeholders representing the main public entities in the Egyptian healthcare sector attended the workshop. They discussed different aspects related to the biosimilars policy framework. The attendees came from the following healthcare sectors: the Health Insurance Organization (HIO), the Egyptian Authority for Unified Procurement, Medical Supply, and Technology Management (UPA), the Universal Health Insurance Authority (UHIA), the Ministry of Health and Population (MoHP), the Egyptian Drug Authority (EDA), University hospitals, and the Egyptian parliament health affairs committee. The workshop attendees’ responses are presented in Table 1.

**Table 1:** Experts' responses to the survey

Domains and questions	Percentage of experts advocating
Domain 1: the overall perception of biosimilars	
Biosimilar medicines:	(*N* = 16)
*a. Have the potential to increase patient access to biological medicines*	**100%**
b. Do not increase patient access to biological medicines	0%
c. Reduce patient access to biological medicines	0%
Biosimilar medicines:	(*N* = 16)
a. Are usually less effective than originators	6%
*b. Are usually equally effective to originators*	**69%**
c. Have the potential to be more effective than the originator	25%
Switching patients from biological medicines to biosimilar alternatives	(*N* = 16)
a. Is associated with a significantly increased risk of immunogenicity	12.5%
*b. Is not associated with an increased risk of immunogenicity in case of a single switch under medical supervision*	**56.25%**
c. Is not associated with an increased risk of immunogenicity even if the patient is switched multiple times	25%
d. Is not associated with an increased risk of immunogenicity even if the patient is switched multiple times without medical supervision	6.25%
Domain 2: pricing	
How much mandatory discount from the originator price would you recommend for the first biosimilar?	(*N* = 16)
a. No mandatory discount	6.25%
b. Mandatory 10% discount	6.25%
c. Mandatory 20% discount	12.5%
*d. Mandatory 30% discount*	**44%**
e. Mandatory 40% discount	31%
How much mandatory discount from the price of the current reference biological product would you recommend for subsequent biosimilars?	(*N* = 13)
a. No mandatory discount	8%
b. Mandatory 5% discount	8%
*c. Mandatory 10% discount*	**38%**
d. Mandatory 15% discount	23%
e. Mandatory 20% discount	23%
Do you suggest a revision of the biosimilar prices periodically?	(*N* = 15)
a. No, the price revision should only happen with the launch of a new biosimilar alternative	20%
b. Yes, every 6 months	0%
c. Yes, every 1 year	27%
*d. Yes, every 2 years*	**53%**
e. Yes, every 3 years	0%
Domain 3: reimbursement	
What do you think the role of Health Technology Assessment (HTA) would be for biosimilars?	(*N* = 16)
a. No HTA is required	0%
*b. HTA is required only when the biosimilar manufacturer intends to extend the reimbursement indication (Patient population) compared to the originator*	**81%**
c. HTA is always required	19%
If a biosimilar manufacturer offers a 50% discount on the originator price, do you agree to provide immunity against removal from the formulary list?	(*N* = 15)
a. No	20%
b. Yes, immunity should be offered for 1 year	27%
*c. Yes, immunity should be offered for 2 years*	**33%**
d. Yes, immunity should be offered for 3 years	20%
How the more expensive biological medicine/s should be disincentivized by healthcare payers?	(*N* = 15)
a. Exclude from the formulary	13%
*b. Can be prescribed only as second-line therapy in the therapeutic guidelines*	**60%**
c. Can be prescribed with a high co-payment (internal price referencing)	27%
What should be the exclusion rule for more expensive biological products?	(*N* = 16)
a. 10% price differential	12.5%
b. 20% price differential	18.75%
*c. 30% price differential*	**31.25%**
d. 40% price differential	18.75%
e. 50% price differential	18.75%
Should we extend the financing protocol to all biologicals within the same therapeutical group with similar efficacy and safety (e.g., erythropoietin, TNF alfa medicines in rheumatoid arthritis) in a way that is more expensive biologicals can be prescribed only after a cheaper alternative fails for any reason?	(*N* = 16)
a. Yes	44%
*b. No*	**56%**
What type of monitoring is applied for prescribing patterns against the financing protocol?	(*N* = 17)
a. No monitoring is applied for prescribers to adhere to financing protocols	0%
b. Prescribing practices are monitored against financing protocols only in case of signals of extreme prescribing practice	12%
*c. Prescribing practices are proactively & routinely monitored against financing protocols*	**88%**
In case of deviation from the financing protocol, should financial disincentives be applied to prescribers?	(*N* = 18)
*a. Yes*	**94%**
*b. No*	6%
Domain 4: biosimilars uptake	
Which measures should be applied to enhance prescribers’ acceptance of biosimilars? Choose all that apply*	(*N* = 17)
*a. Generate real-world evidence about biosimilars*	**88%**
b. Conduct a systematic literature review on the immunogenicity related to switching patients to biosimilar alternatives	64%
c. Developed clinical guidelines about switching	60%
*d. Introduce financial protocol to advocate the first-line use of biosimilars for *de novo* patients*	**82%**
e. Mandate to switch patients on chronic biological treatments to cheaper biosimilar alternatives	47%
f. Share information on how biosimilar adoption decreases the pharmaceutical expenditure	60%
*g. Share information on how biosimilar adoption improves patient access to biological medicines*	**94%**
h. Disseminate results of cost-effectiveness studies comparing biosimilars and originators	70%
i. Others (please specify)	2.1%
Which of the following aspects should be addressed with the patients to enhance their acceptance of biosimilars?*	(*N* = 18)
a. No need for patients’ education about biosimilars	5%
b. Explanation of decreased cost	44%
*c. Using co-payments for biological medicines with a higher cost*	**61%**
d. Full price should be paid by patients for biological medicines with a significantly higher cost	11%
*e. Better access should be explained—treating more patients from the same budget, earlier initiation of biological treatment or longer treatment* duration	**94%**

Experts were asked to vote for each question. The number of respondents to each question is indicated next to it. The percentages represent the number of experts advocating this response divided by the total voters of the question. In questions where experts were allowed to choose more than one answers, percentages sum more than 100%

Bold indicated the highest percentage of votes within the options of the question. In case of multiple-choice questions, more than one option were highlighted if the values were close.

Italics it indicates the option with the highest percentage of votes

^*^Experts were allowed to choose multiple answers

#### Survey domain 1: overall perception of biosimilars

There was a consensus among the experts on biosimilars’ potential to increase access to biological medicines. Regarding efficacy, the majority of experts (94%) believed that biosimilars are usually equally effective to the originators or could even be—in some cases—more effective. Only one expert responded that biosimilars are usually less effective than the originators.

Concerning switching from originators to biosimilars, immunogenicity was the utmost consideration. Most experts (88%) believed that biosimilars are not associated with a significant increase in immunogenicity. Fifty-seven percent believed that single switching under medical supervision is not associated with a significant risk of immunogenicity. In contrast, 31% were more liberal and believed switching is not associated with a significant risk of immunogenicity, even if it occurred multiple times. Only 12% believed that switching is associated with a significantly increased risk of immunogenicity.

#### Survey domain 2: pricing

The survey addressed three aspects of pricing: pricing of the first biosimilar, pricing of the subsequent biosimilars, and the periodic revision of the biosimilars' prices.

The majority of experts (94%) believed that a mandatory discount from the originator’s price should be applied when the first biosimilar is introduced. Most of them (75%) advocated that the price of the first biosimilar should be discounted by 30–40% of the originator's price, with a median discount value of 30%. Others suggested that the discount should only be 10–20%. As for pricing subsequent biosimilars, 92% of the experts believed that subsequent biosimilars’ prices should be discounted compared to the first biosimilar’s price. They suggested a discount of 5%-20% with a median discount value of 10%.

Eighty percent of the experts suggested revising the biosimilars' prices periodically. However, a consensus was not reached at the time of revision. Some suggested annual revisions, and most of them suggested biennial revisions (every 2 years). Nevertheless, 20% of them recommended price revision only at the launch of new biosimilars.

#### Survey domain 3: reimbursement

Concerning the reimbursement of biosimilars, the following were discussed: the role of HTA and the immunity for a biosimilar against removal from the formulary and applying financing protocols. Financing protocols are mandatory clinical guidelines developed jointly by clinical societies and payers, which considers both clinical and economic aspects.

For the question asking whether HTA is required for biosimilars, the vast majority (81%) regarded it as mandatory if the manufacturer intends to extend the reimbursement indication compared to the originator. However, only a few experts recommended mandatory HTA in all reimbursement decisions.

Since the experts recommended a mandatory discount for introducing the first biosimilar (30% discount of the originator’s), most experts (80%) recommended that this first biosimilar product should be immune to removal from the formulary if its price is at least 50% less than the originator. The immunity privilege means that if a lower-price biosimilar were introduced after that, the first biosimilar (the immune) would not be delisted for the whole immunity period. The experts had different opinions regarding the exact period of immunity; opinions varied from one to three years. The recommendation was intended to protect biosimilars that are at least 50% cheaper than the originator from being removed from the formulary, even if a new biosimilar that is 30% cheaper than them is introduced.

All experts endorsed that payers should disincentivize prescribing more expensive biologics after the introduction of biosimilars. The majority (60%) suggested moving them to the second line of therapy as a disincentive. The rest of the experts had other viewpoints, such as excluding expensive biologics from the formulary list (13%) or adding a high co-payment (27%).

There was a consensus among the experts that more expensive biologics (biologics, whether originator or biosimilar, that are more expensive than the reimbursed one) should be excluded from the formulary if their price differential (percentage difference between the price of the currently used biological and the newly introduced lower-price biological) is above a certain threshold. However, they had different views regarding the value of that threshold. Most of them (69%) recommended 30–50%, but 23% of the experts recommended a 10–20% price differential.

The majority (56%) voted against extending the financing protocol to all biologics within the same therapeutic group so that more expensive biologics can be prescribed only after cheaper alternatives fail.

Most experts (88%) believed that prescribing practices should be proactively and routinely monitored for following financing protocols with financial disincentives applied in case of deviation from the financing protocols. For example, physicians and healthcare facilities may have their payments reduced if they deviate from the financing protocols.

#### Survey domain 4: biosimilars uptake

Biosimilars uptake is the diffusion of biosimilars within the healthcare system. This step is crucial for the subsequent success of biosimilar policy implementation, and it mainly depends on prescribers' and patients’ acceptance.

To increase patients’ acceptance of biosimilars, experts were given multiple options to choose from, and they were allowed to select multiple strategies. The strategies most advocated by the experts were explaining how biosimilars provide better access to expensive biologics (94%) and using co-payments for the expensive biologics (61%).

As for enhancing prescribers' acceptance of biosimilars, experts were given multiple options to choose from, and they were allowed to select multiple strategies. The strategies most advocated by the experts were sharing information about increased access by adopting biosimilars (94%), generating real-world evidence about biosimilars (88%), introducing financial incentives for the first-line use of biosimilars for naïve patients (82%), and disseminating the results of cost-effectiveness studies comparing biosimilars and originators (70%). Financial incentives may include gain-share agreements, where part of the savings can be shared with the prescribing centers or the prescribers [21].

#### Other discussions during the workshop

There were several side discussions and related topics that popped out during the workshop. These were very useful and helped in shaping the final recommendations. Other than those topics that were addressed in the survey questions, the workshop attendees discussed biosimilars’ post-marketing policies and pharmacovigilance. They recommended providing incentives for the manufacturers to establish registries and conduct real-world evidence studies of biosimilars. These data sources could help regulators follow up on the consequences and patterns of biosimilar uptake and modify policies accordingly. Moreover, they discussed initiating a pharmacovigilance (PV) framework specific to biosimilars and biologics rather than depending on the already-existing pharmacovigilance framework. In Egypt, the market authorization holders (MAH) should have a full-time Qualified Person Responsible for Pharmacovigilance (QPPV). The MAH should perform routine pharmacovigilance (the primary/minimum set of activities required for all medicinal products and should be implemented for all safety concerns). These routine activities include the preparation of a Periodic Benefit Risk Evaluation Report (PBRER), adverse events reporting, and continuous monitoring of the efficacy and safety profile. According to the guidelines, the MAH may establish more than one pharmacovigilance system, e.g., specific systems for particular types of products (e.g., vaccines).

Based on the stakeholders’ responses to the survey and the discussions during the workshop, recommendations for the biosimilar policy framework in Egypt were summarized and are presented in Table 2.

**Table 2:** Summary of the recommendations for the biosimilars’ policy framework in Egypt

Domain	Framework policy recommendation
Pricing	Mandatory discount of 30% for the first biosimilar from the originator's price
	For subsequent biosimilars (2nd or later), a 10% discount should be applied from the preceding biosimilar introduced
	Biosimilars' prices should be revised at pre-specified intervals, annual or every two years
Reimbursement	HTA, specifically CEA & BIA, should be used when the manufacturer applies for extending the reimbursed indication compared to the originator
	Immunity for one year or more against removal from the formulary should be provided for biosimilars that offer at least a 50% discount compared to the originator
	Prescribing practices should be proactively and routinely monitored against financing protocols. Furthermore, in case of deviation from the financing protocol, financial disincentives should be applied to prescribers
	Switching of existing patients should be done under medical supervision
	More expensive biological medicine/s should be disincentivized by health care payers, such as moving them to the second line of therapy
	More expensive biological products should be excluded from the formulary if they fail to reduce the price gap below 30%
Biosimilars’ uptake	Share information with patients & prescribers’ on how biosimilar adoption improves patient access to biological medicines. Better access should be explained—treating more patients from the same budget, earlier initiation of biological treatment or longer treatment duration
	Introduce financial protocols to advocate the first-line use of biosimilars for de novo patients by prescribers
	Generate real-world evidence about biosimilars to address safety and effectiveness concerns

Each domain in the table includes several recommendations for an efficient biosimilars framework based on the survey results and experts’ discussions

## Discussion

Healthcare policymakers aim to maximize health gain while minimizing costs and maintaining financial sustainability [36, 37]. Some limited-budget countries restrict access to costly biologics to contain pharmaceutical spending. Biosimilars, which are comparable to innovative biologics but at lower prices, can provide a solution [38, 39]. However, there is a need for a clear regulatory framework for pricing, reimbursement, and clinical uptake in many lower-middle-income countries, including Egypt [16]. Our study sought to fill this gap by seeking experts' recommendations.

The main recommendation of the experts included pricing biosimilars according to the internal reference pricing, revising their prices annually or biennially, applying HTA to biosimilars only in certain situations, reimbursement decisions, immunizing the biosimilars against removal from formulary under certain conditions, applying financing protocols with incentives and disincentives upon violation, and sharing information with prescribers and patients to enhance biosimilars uptake.

### Overall perception about biosimilars

Concerning the benefits of biosimilars, biosimilars are considered a way to increase patients' access to biologics without compromising clinical efficacy and safety outcomes. This agrees with the perception of payers and clinicians reported in the literature [5, 40]. Therefore, such views may encourage biosimilars' adoption and diffusion in the healthcare system.

As for the postulated risk of immunogenicity, switching to biosimilars was not considered a significant risk of immunogenicity, especially if it is a single switch that is conducted under medical supervision. This agrees with the body of evidence from both controlled trials [41–44] and real-world evidence [41, 43, 44]. Such beliefs and associated evidence should be communicated with prescribers to address their concerns about the safety of biosimilars to encourage them to use biosimilars for their patients, especially in countries where the need for them is high, and biosimilars’ use is still uncommon.

### Pricing

As for the pricing scheme of biosimilars, a mandatory 30% discount for the first biosimilar from the originator's price was recommended. This internal reference pricing system is applied in some countries [19, 20], such as Spain, Austria, Italy, Switzerland, and Saudi Arabia [45]. Furthermore, for subsequent biosimilars, applying a 10% discount to the price of the previously introduced biosimilar was advocated. This pricing policy also is applied in some countries [29], such as Saudi Arabia [45], Austria [19], and Hungary [46]. Applying such a pricing policy intends to sustain the funding of such expensive medications for a broader segment of patients. Also, it will create a competitive environment among the manufacturers for the benefit of the patients and the healthcare system.

The drug market, especially biologics, is expanding dynamically. New molecules are introduced frequently. Therefore, re-evaluation of biosimilars’ prices on a regular basis was recommended, either annually or biennially. This price review policy is implemented in some countries, such as France, where the prices of both biologics and biosimilars are revised every 18–24 months based on the penetration rate of the biosimilar [47, 48]. Such pricing policy for biosimilars is expected to help the system achieve the goal of healthcare sustainability and optimize pharmaceutical expenditure.

### Reimbursement

Since economic evaluation is necessary for healthcare systems, HTA for biosimilars was recommended as a mandatory requirement only if the manufacturer applies for extending the reimbursement indication compared to the originator. This is close to the policy applied in Finland, where economic evaluation is required if a drug contains a new active pharmaceutical ingredient or the indication for the reimbursement status is going to be significantly expanded [33]. Worldwide, countries' policies vary regarding the role of HTA. HTA review follows the same process for new drugs [49] in Canada, New Zealand, and Australia. Similarly, in Estonia, full HTA is necessary for the biosimilar regardless of the status of the reference medicine [50]. Some countries do not require HTA if the reference product is reimbursed, such as Scotland [51], Slovakia [52], and Poland [53]. In Wales [54], biosimilars are not appraised if the reference medicine is accepted for the same population for the same indication and in case the cost of the biosimilar does not exceed the reference medicine. In Finland, Germany, and the Netherlands, HTA review is not routinely required [49]. Countries design their HTA regulations for biosimilars based on their financial status and objectives of biosimilars’ integration into the healthcare system. We believe that in LMIC (low-middle-income countries), where HTA is not widely implemented biosimilars should not be a priority when it comes to HTA implementation as those countries do not have the capacity [55]. Furthermore, considering limited resources to conduct HTA high budget impact innovative pharmaceuticals should be the priority when implementing HTA [37, 55–57].

For the status of the biosimilar in the reimbursed formulary, it was recommended to provide biosimilars immunity against removal from the formulary if their price is at least 50% of the originator’s price as an incentive for pharmaceutical companies to reduce the price of their biosimilars by 50% instead of the obligatory 30%. Such immunity can be provided for 1 year or more. This is close to the single-winner policy in tenders in some countries. For example, in some countries like France and Denmark, the single-winner system is applied with a 12-month contract that is renewable in France once or twice [30]. In Hungary [30], bids are submitted twice a year, and winners gain preferred provider stays during the next 6 months. This policy aims to encourage biosimilar manufacturers to offer the lowest possible prices; thus, the payer can accomplish better financing efficiency.

It was advocated to move the more expensive biologic to the second line of therapy in the prescribing guidelines of biosimilars. This strategy is applied in other countries [8, 24], such as Sweden [58], Italy [59], and Norway [60]. Also, applying high co-payments was suggested to discourage the patients from using the more expensive biologics and encourage the wider use of cheaper biosimilars. This strategy has been adopted in some countries, such as Germany, Hungary, Poland, Spain, and Sweden [24, 61].

Delisting the more expensive biologics was recommended if the price gap between them and cheaper alternatives reached 30% or more. Such a strategy is applied in Hungary; drugs with a price that is 30% higher than that of the reference products (defined as the cheapest one on the list) are removed from the reimbursement list (i.e., positive list) 4 months after the biosimilar bid [29]. This policy may encourage the manufacturers of the more expensive biologics to offer price discounts and the manufacturers of the cheaper biosimilars to decrease the prices accordingly when the prices are reviewed periodically. Additionally, this will enhance the sustainability of access to biologics by ensuring reimbursing affordable prices.

### Biosimilars uptake

Increasing biosimilar uptake requires enhancing the stakeholders' acceptance (the physicians and the patients). Relying only on free-market forces may not be enough, mainly due to the exaggerated theoretical concerns about biosimilars' evidence base and the fear of immunogenicity and decreased efficacy. That being the case, there was a recommendation to communicate educational materials and events with prescribers on how biosimilars can enhance patient access to innovative medicines. Some countries apply similar policies. For example, in the United Kingdom (UK), success stories about cost savings from biosimilars have been published by some hospitals [62].

Introducing financial protocols was recommended in order to encourage physicians to use biosimilars as the first line for de novo patients. Financial incentives are used in the UK, and a gain-share agreement has been established with considerable mutual benefits [63]. Moreover, different financial incentive schemes have been implemented in other countries, such as France, Germany, Italy, and the United Kingdom [64].

Patients' acceptance of biosimilars is substantial in patient-centered healthcare. Within this context, there was a recommendation about initiating educational campaigns for patients to raise their awareness about the benefits of accepting biosimilars. Also, applying co-payments to patients for expensive biologics was suggested. The co-payments are applied in different countries like Germany and Sweden [61]. For example, in Sweden, if the original medicine is chosen instead of a biosimilar, patients have to cover the price difference between the reference medicine and the biosimilar. Also, there was a recommendation for educating patients on how biosimilars may enhance their access to expensive and innovative medications and improve their health. This strategy has improved patients' acceptance in different studies [65, 66].

To enhance the diffusion of biosimilars, proactive and routine monitoring of physicians' prescription practices was recommended to ensure that prescribing patterns do not exceed the assigned quotas. In case of a deviation from these protocols, applying financial penalties was recommended. This comes in agreement with policies applied in various countries. For instance, close monitoring of biosimilar prescriptions was reported in Belgium, Germany, Italy, and Sweden [24]. Financial penalties for prescribers who are not following quotas or targets were reported in Germany and Italy [67, 68]. In contrast, potential financial rewards for physicians who meet their targets are reported in Italy [69].

It was advocated that authorities provide incentives to the biosimilar manufacturers to establish registries for biosimilar products. This would generate real-world evidence to help clinicians and decision-makers evaluate biosimilars' safety and efficacy in real life. In a survey of 10 European countries, nine countries reported regular assessment of pharmacovigilance data on biosimilars [5]. Additionally, national regulatory authorities in Hungary and Slovakia reassess data on effectiveness after registering a new biosimilar to ensure equal health outcomes with the originator [5].

## Conclusion

In conclusion, the survey results and the workshop show a positive attitude of the stakeholders toward using biosimilars. Moreover, their recommendations mostly come in line with the policies adopted worldwide in high-income and lower-middle-income countries, aiming to enhance patient access while containing healthcare expenditure.

### Limitations

Our study comes with some limitations. Since the introduction of biosimilars is relatively recent in the Egyptian market, there is a lack of consensus among the experts in some areas, such as the percentage of price discounts. Such an agreement can be reached in the near future after the experts gain more real-life experience with biosimilars in local settings. Also, some details about the financial incentives were not discussed. This is also attributed to the limited diffusion of biosimilars in the local market. Another limitation is related to the low number of participants. This is due to the presence of a limited number of experts who have a good understanding of all related topics and would provide valuable inputs for the survey from practical experience. However, we ensured that all the major public healthcare entities in Egypt were represented by experts.

## Supplementary Information

**Additional file 1.** Survey voting results (charts).

## Data Availability

All data generated or analyzed during this study are included in this published article and its supplementary information files.

## Abbreviations

CHE: Current health expenditure
EDA: Egyptian Drug Authority
EIPICO: Egyptian International Pharmaceutical Industries Company
EMA: European Medicines Agency
FDA: Food and Drug Administration
GDP: Gross Domestic Product
HIO: Health Insurance Organization
HTA: Health Technology Assessment
LMIC: Low-middle-income countries
MoHP: Ministry of Health and Population
UHIA: Universal Health Insurance Authority
UPA: The Egyptian Authority for Unified Procurement, Medical Supply, and Technology Management
