# Supplementary material for: Maximizing the benefits of using biosimilars in Egypt
Source: J Pharm Policy Pract. 2023 Jun 26;16:79. doi: 10.1186/s40545-023-00581-w (PMC10291771; doi:10.1186/s40545-023-00581-w)

**Additional file 1: Biosimilars survey voting results (charts)**

# Domain 1: Overall perception of biosimilars


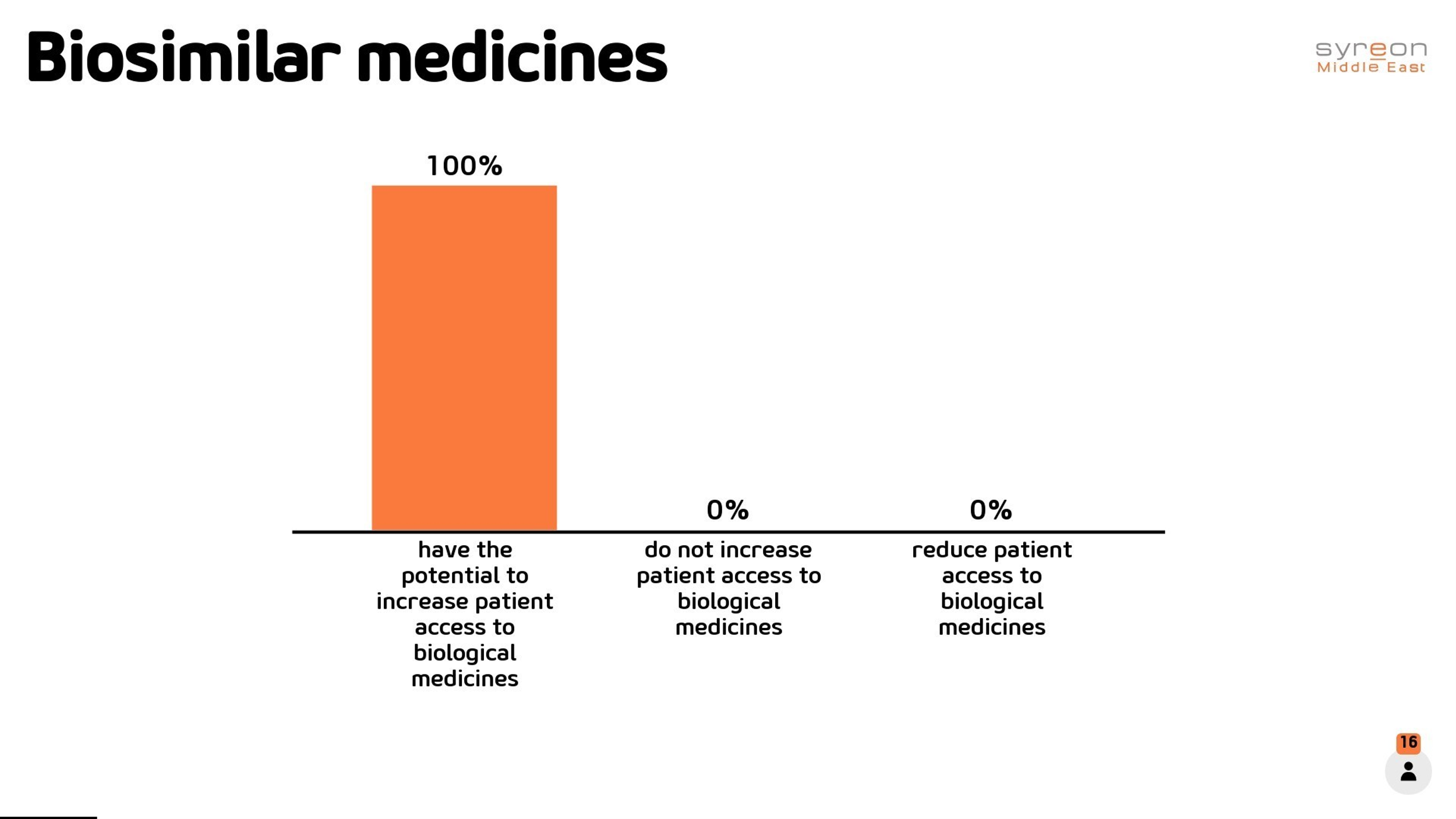


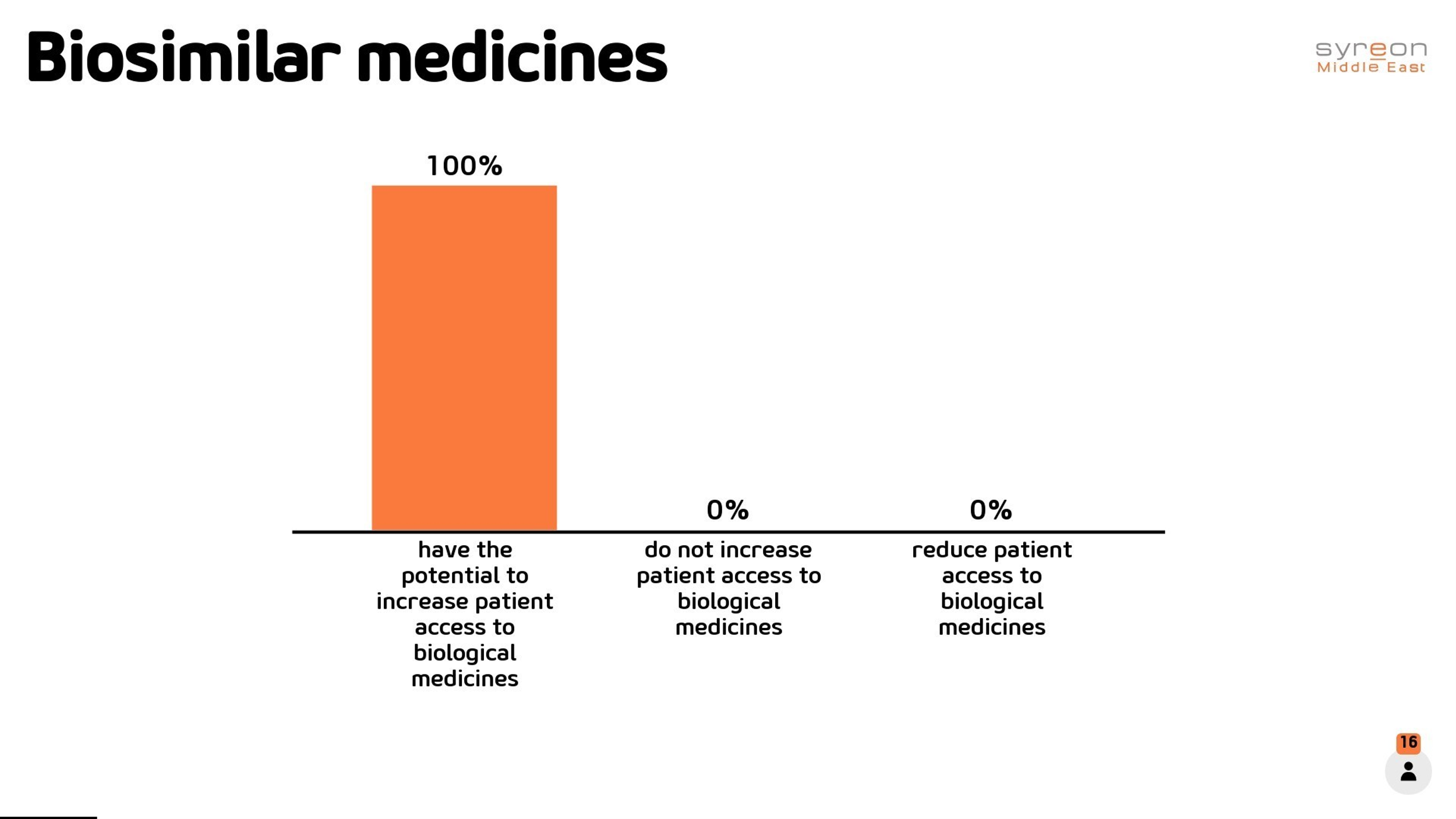


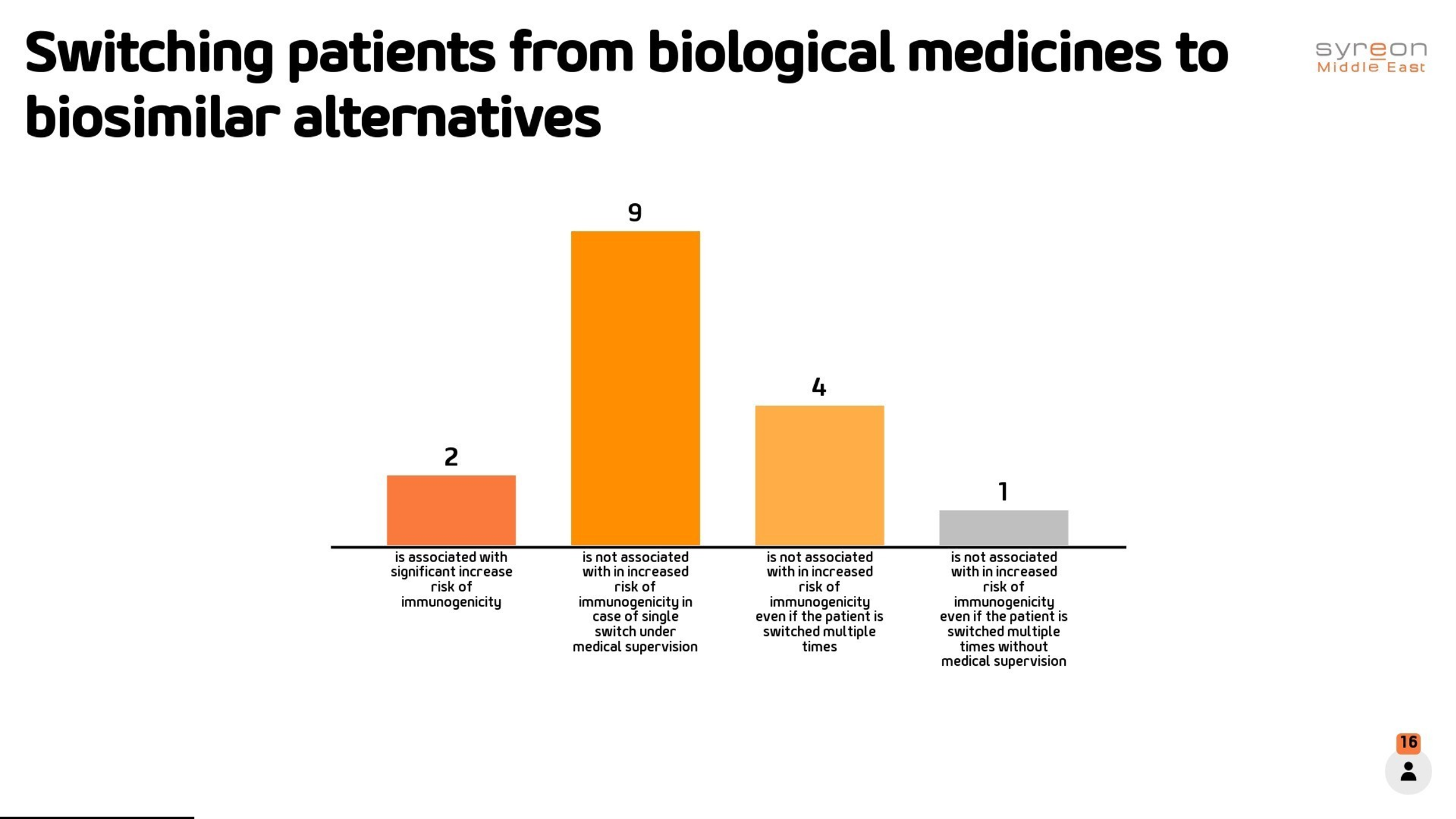


# Domain 2: Pricing


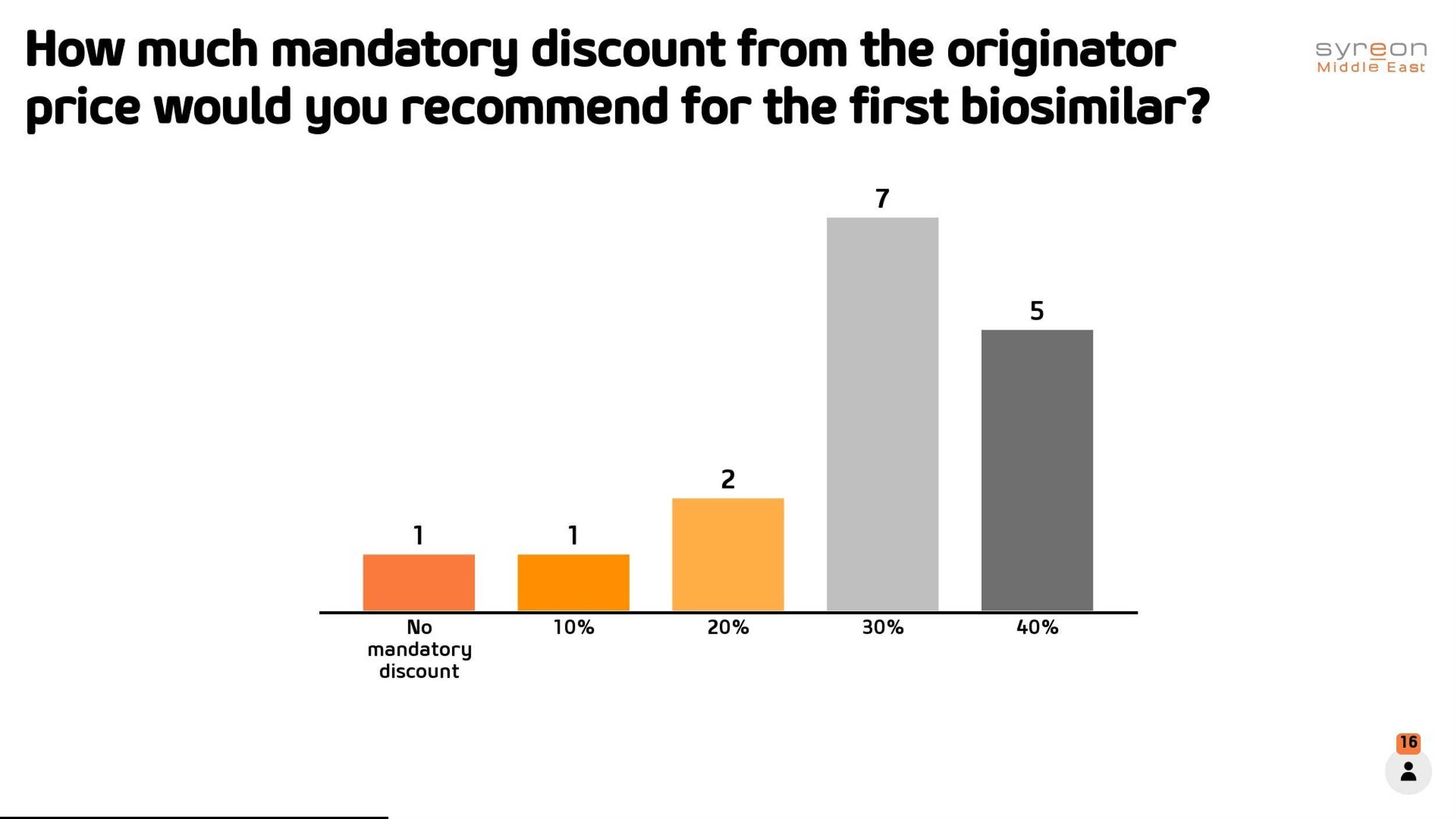


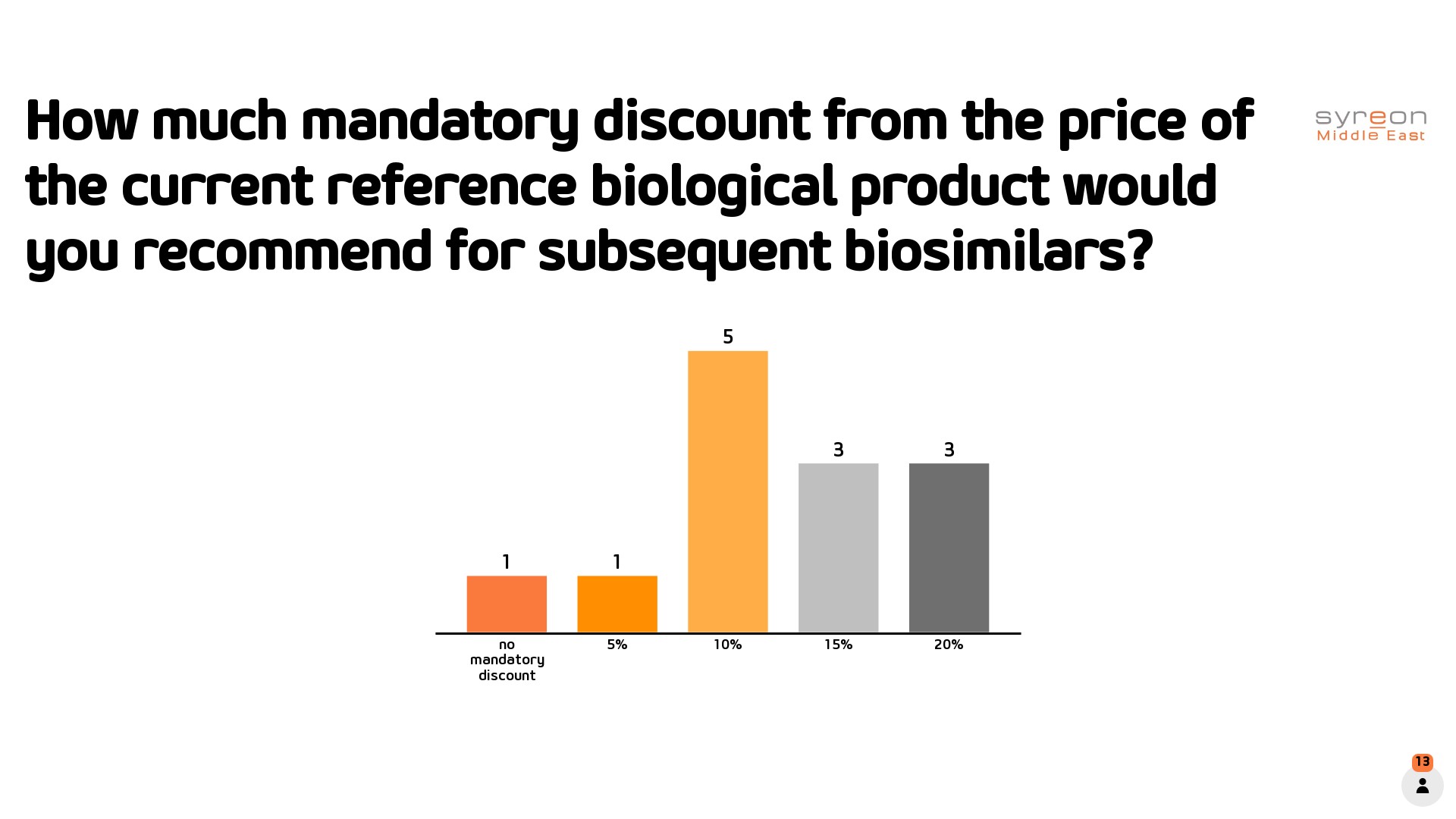


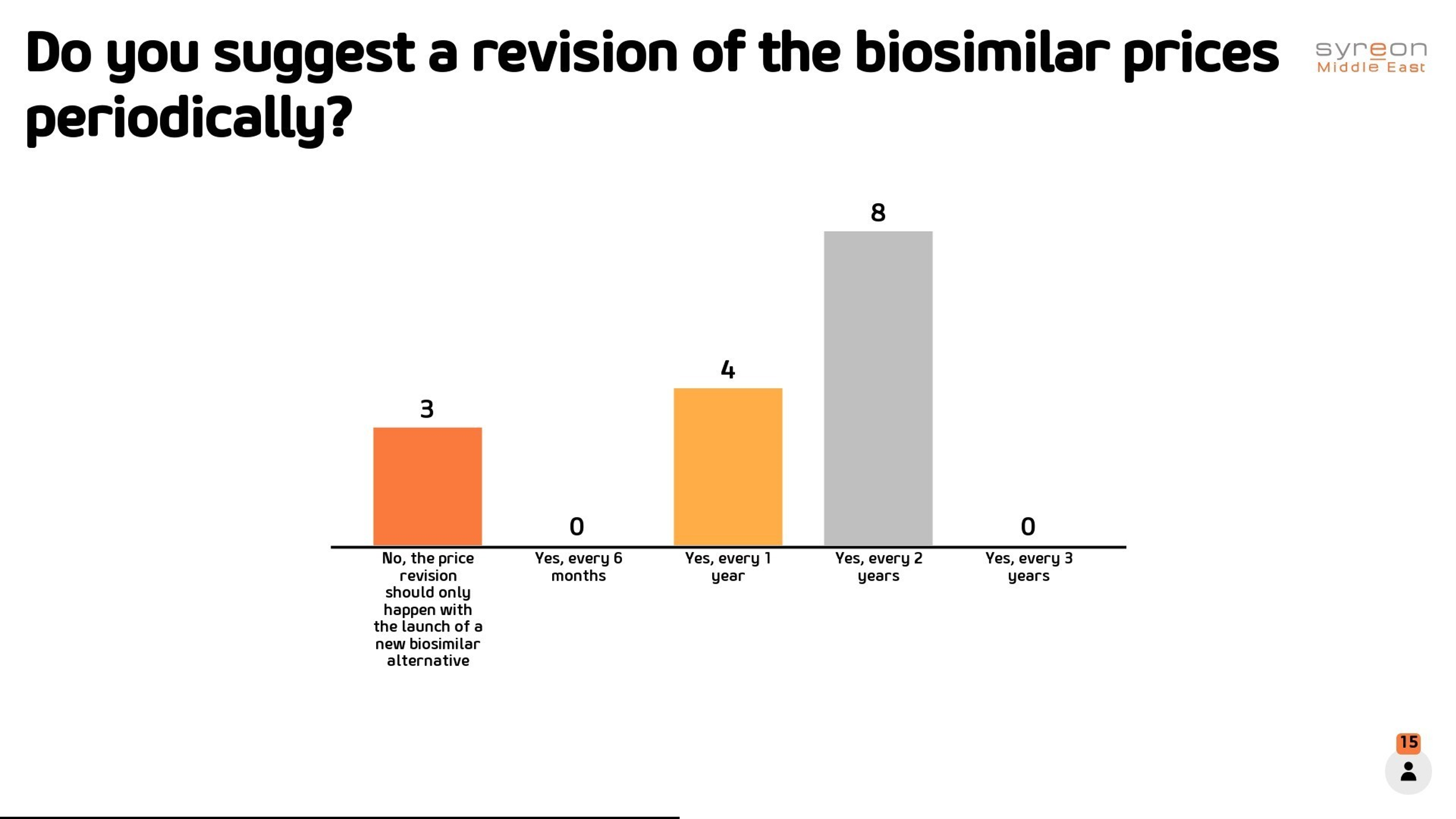


# Domain 3: Reimbursement


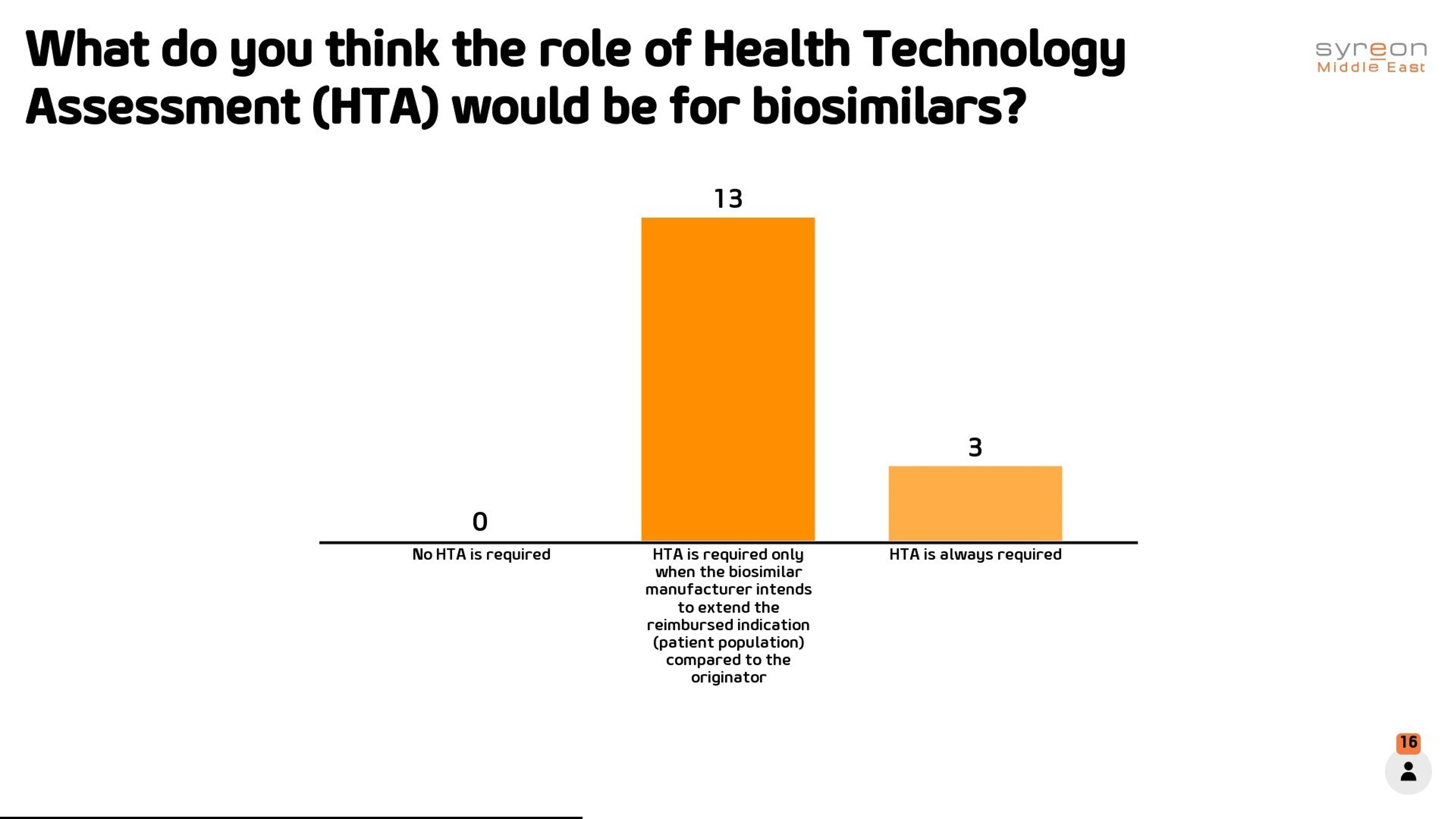


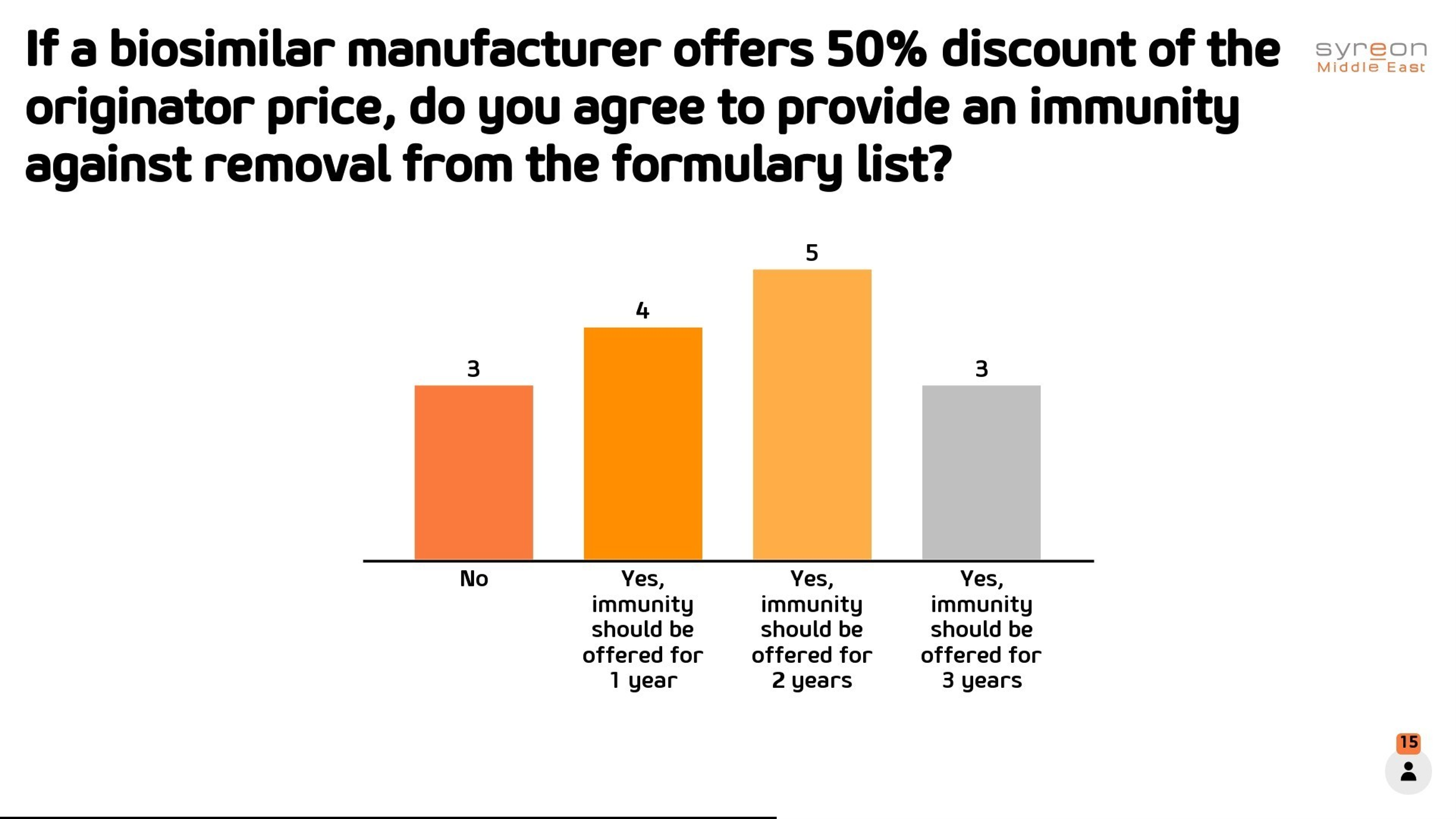


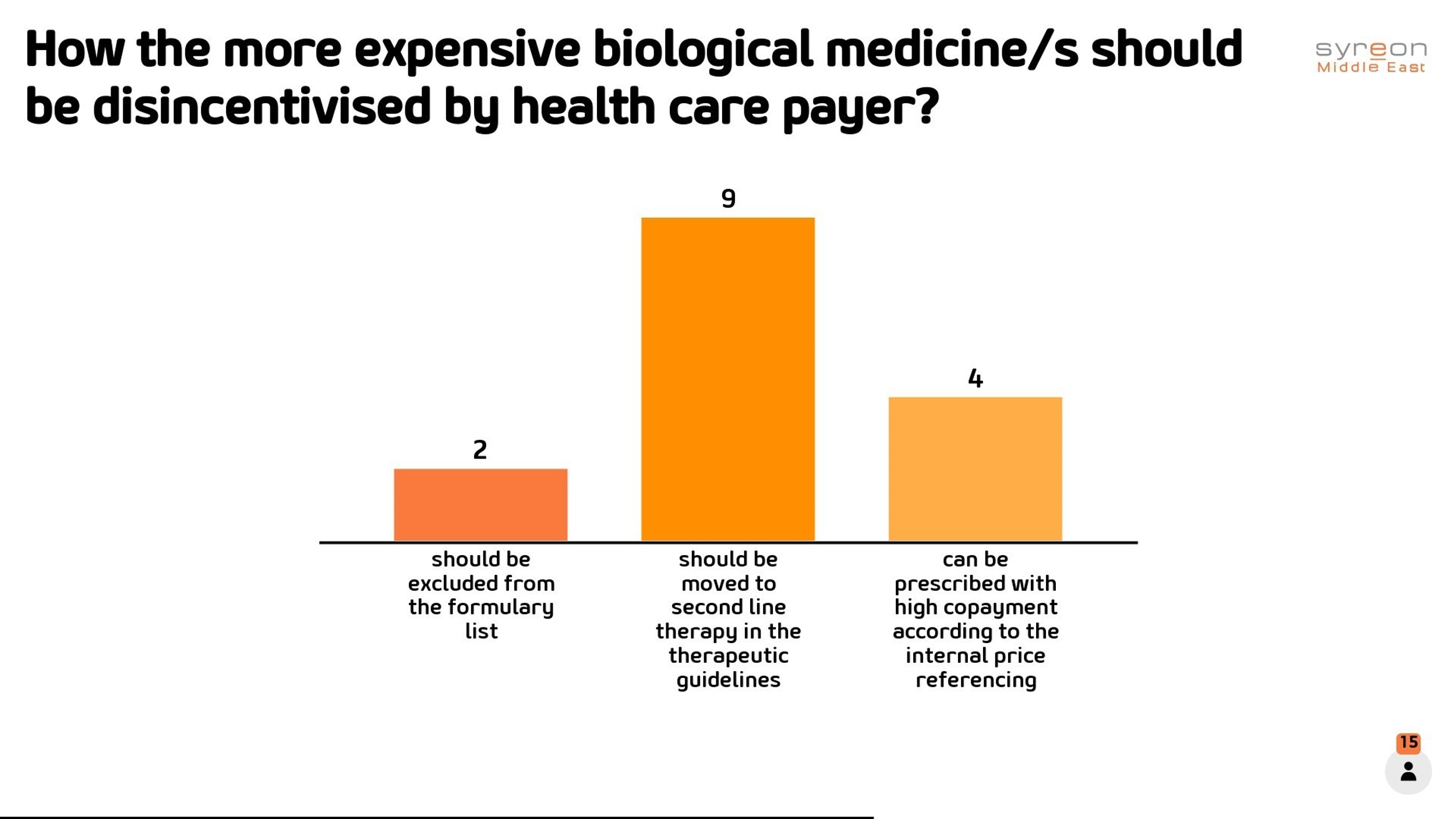

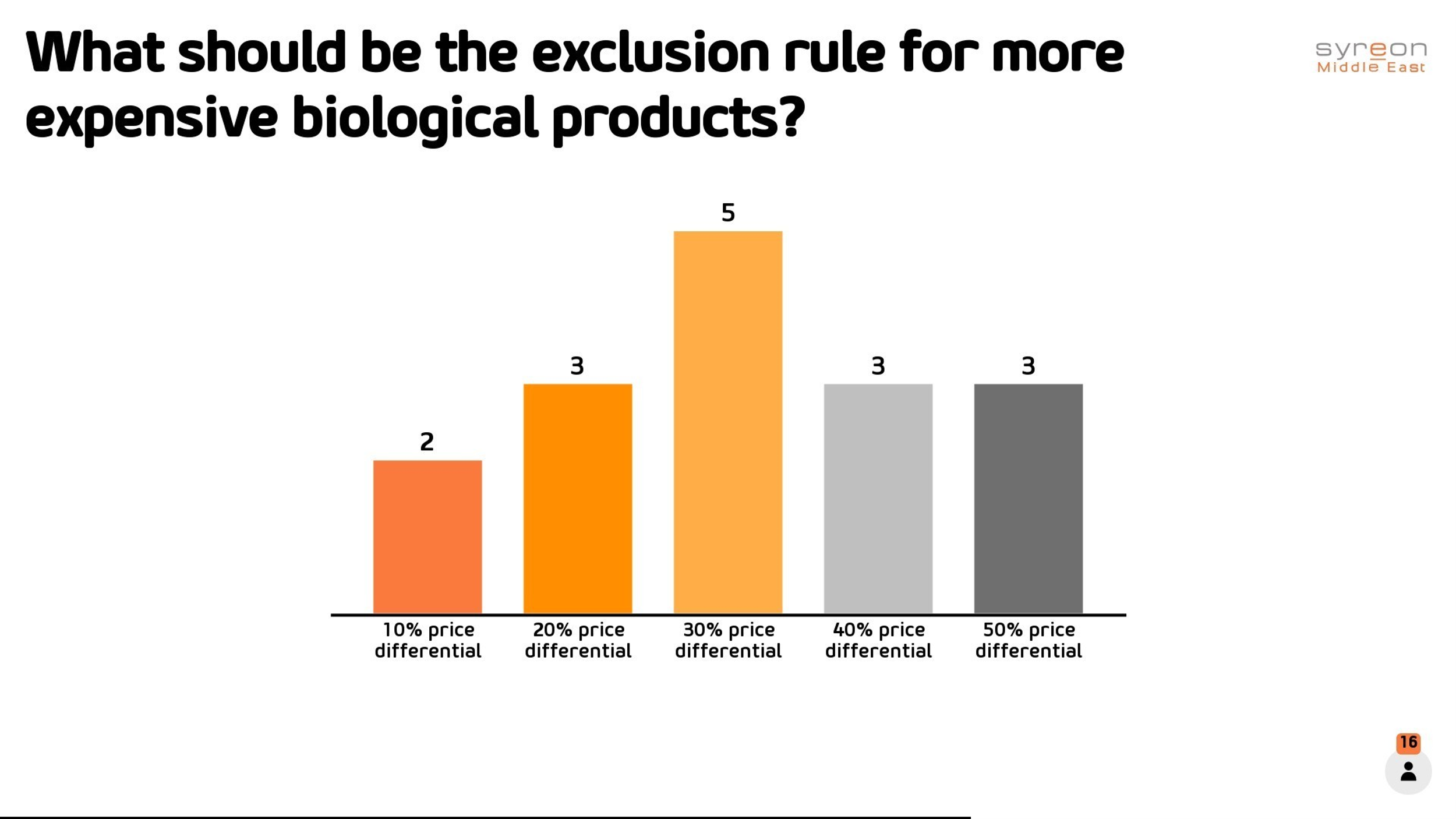


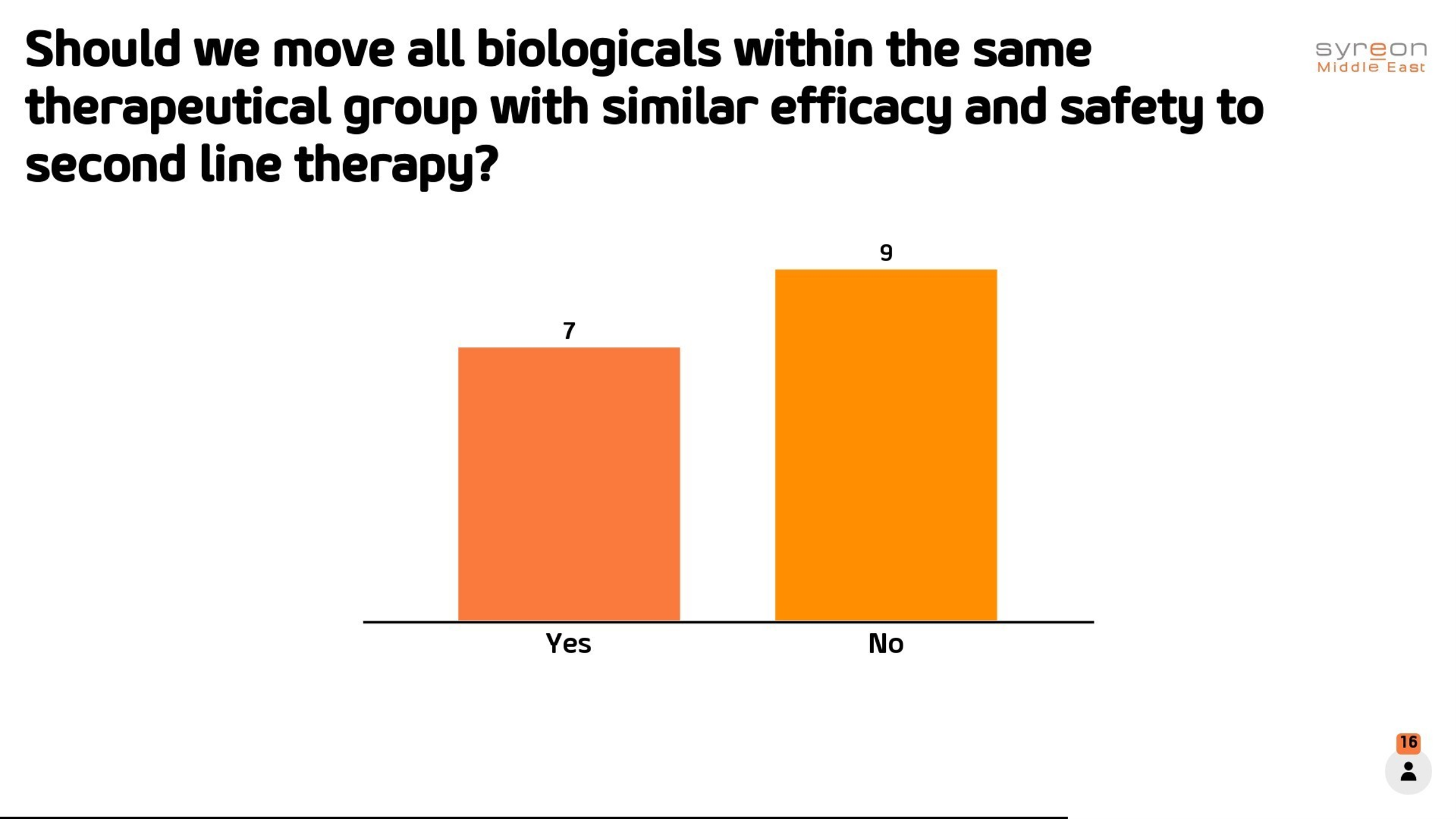


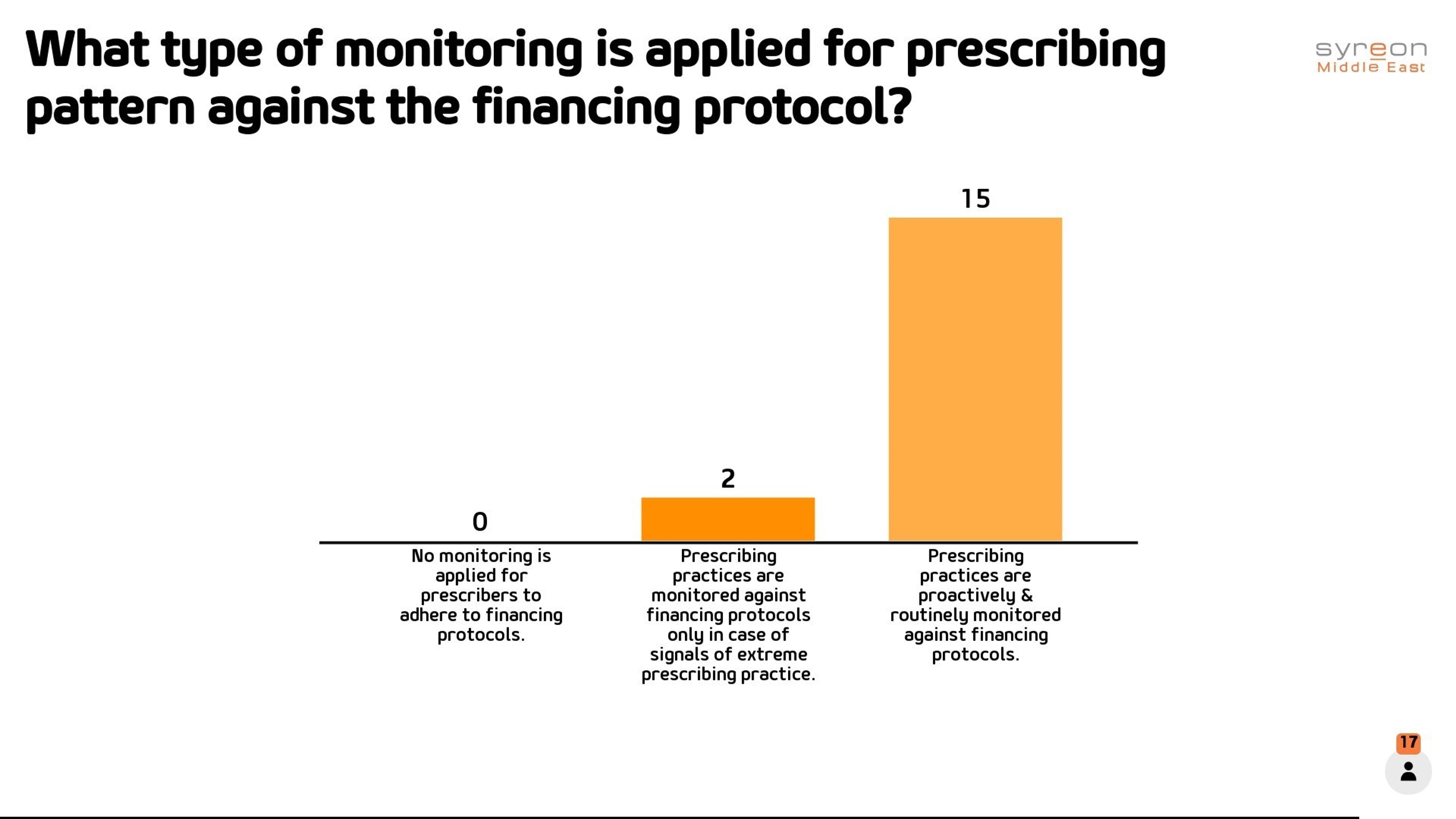


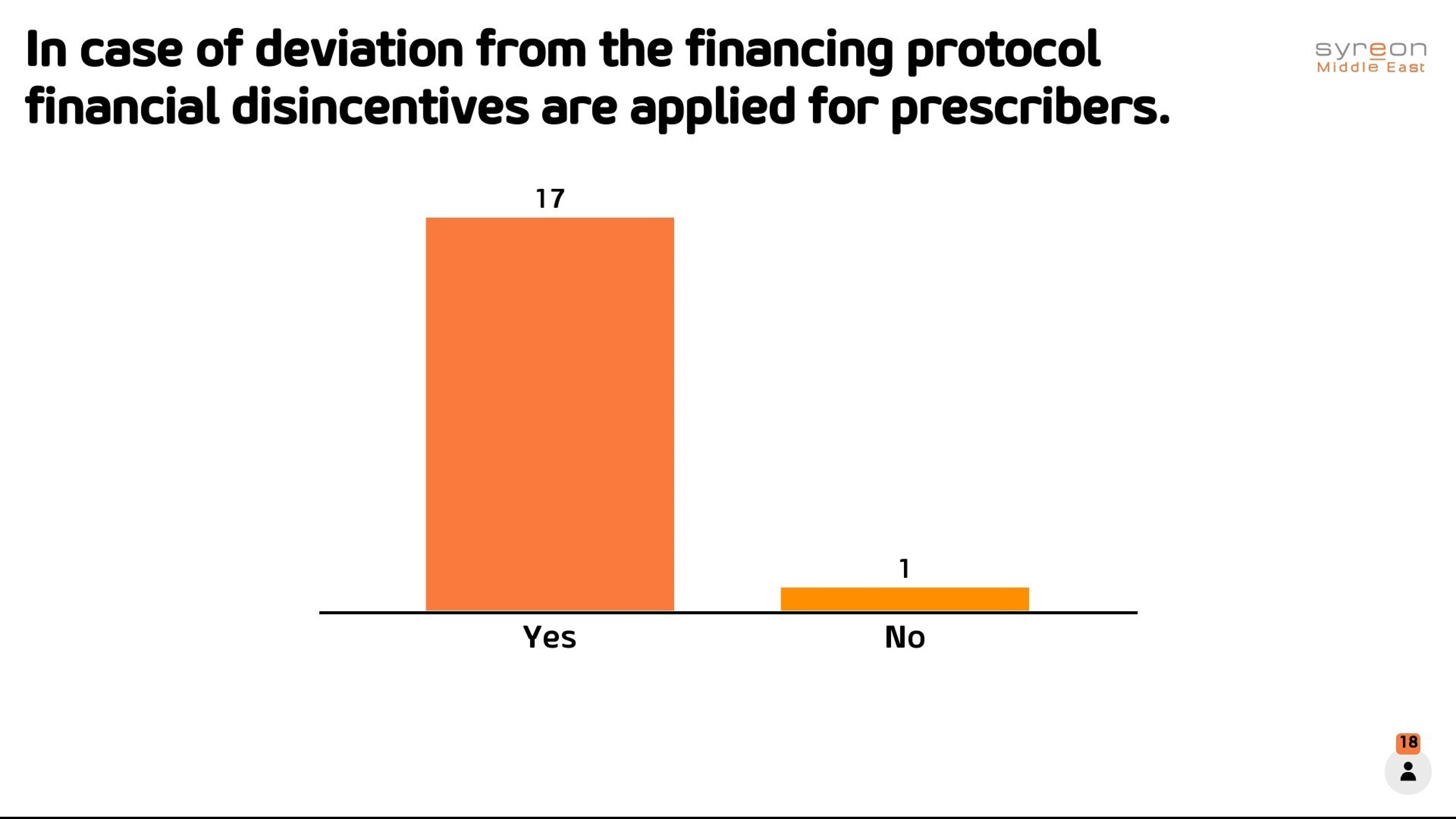


# Domain 4: Biosimilars uptake


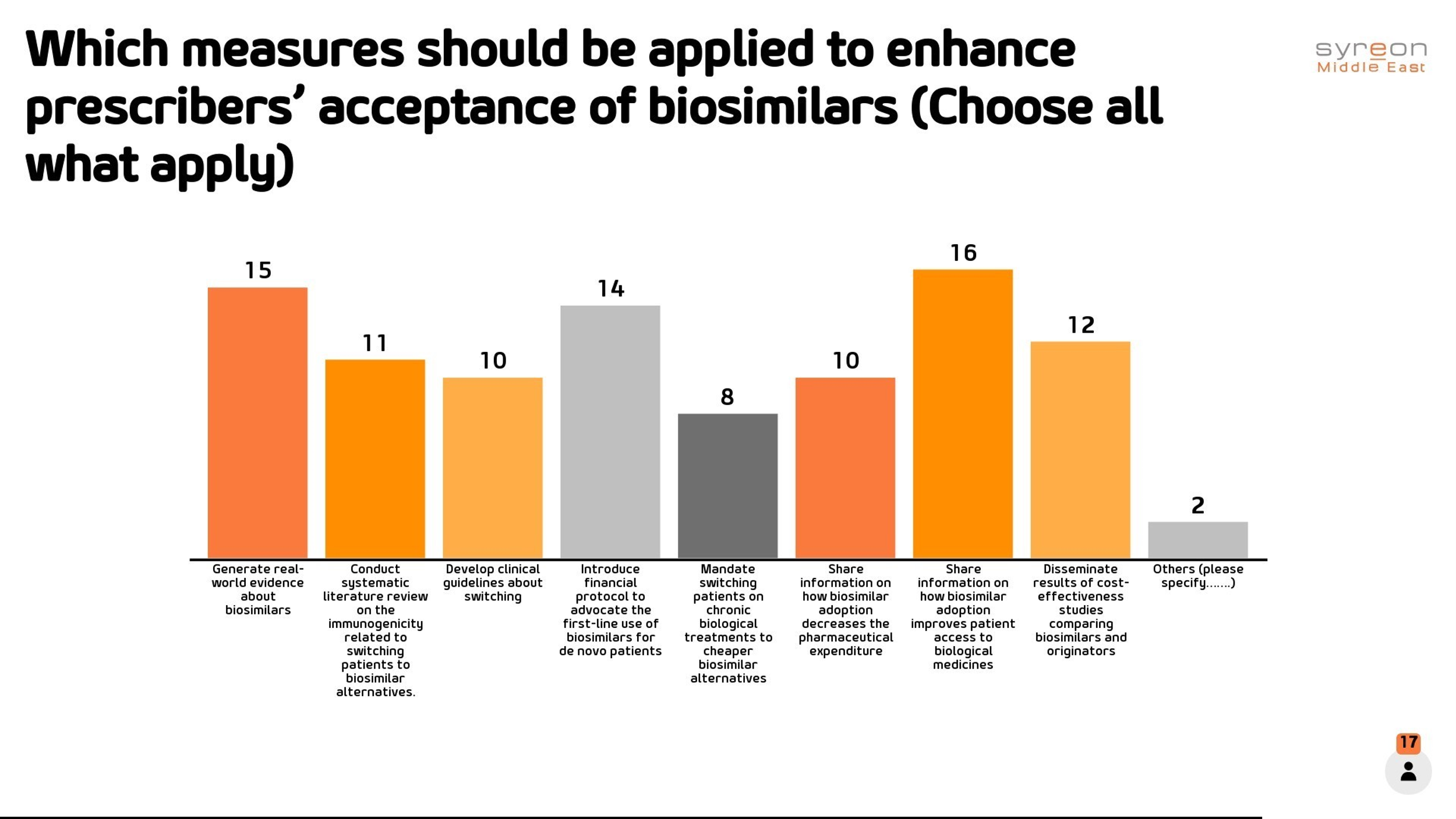


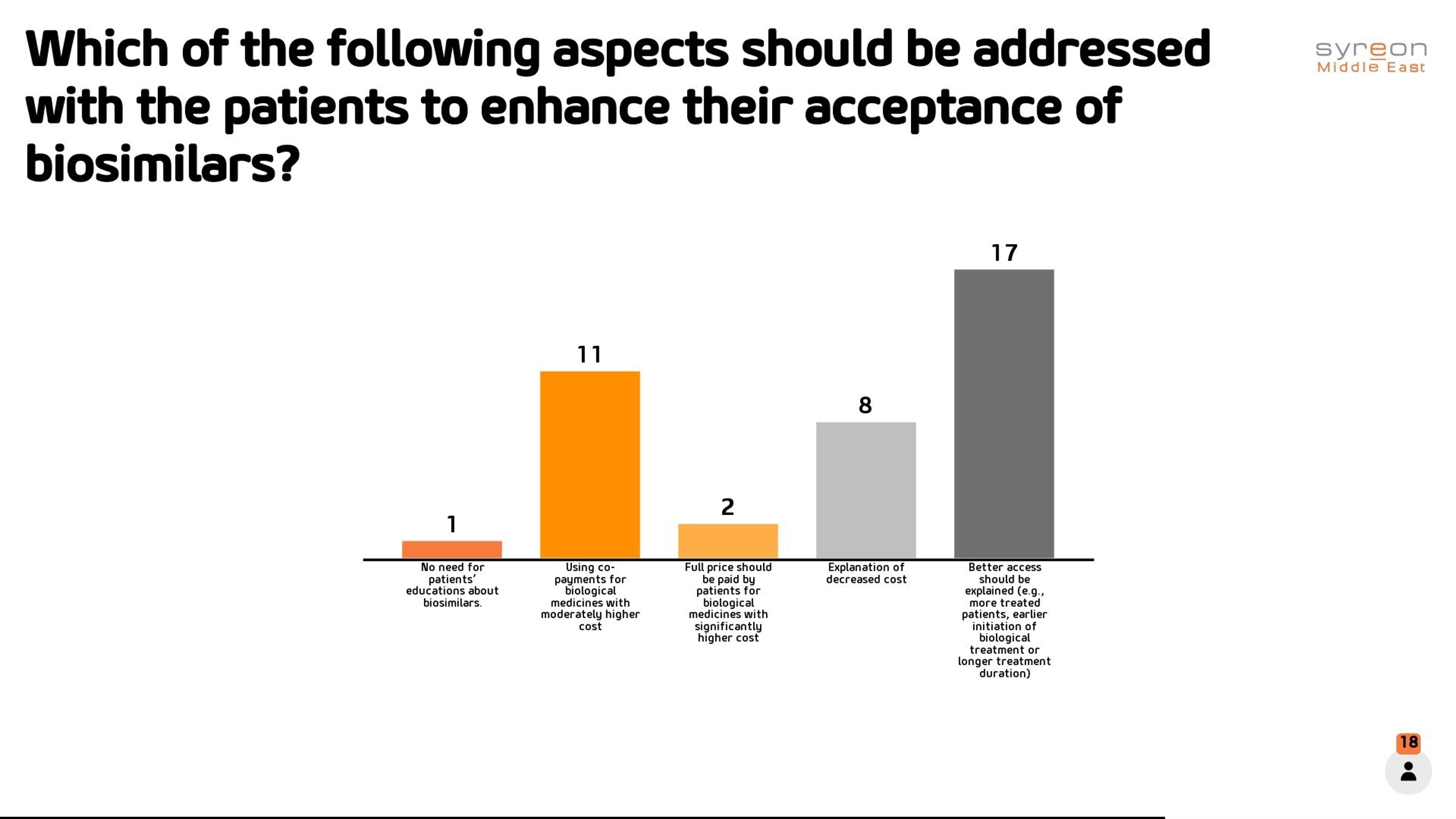

Supplement: Supplementary file 1 — Additional file 1. Survey voting results (charts). [file 40545_2023_581_MOESM1_ESM.docx]
